# Freely distributed bed-net use among Chano Mille residents, south Ethiopia: a longitudinal study

**DOI:** 10.1186/1475-2875-12-23

**Published:** 2013-01-18

**Authors:** Eskindir Loha, Kebede Tefera, Bernt Lindtjørn

**Affiliations:** 1 School of Public and Environmental Health, Hawassa University, Hawassa, Ethiopia; 2 Centre for International Health, University of Bergen, Bergen, Norway; 3 Arba Minch College of Health Sciences, Arba Minch, Ethiopia

## Abstract

**Background:**

A huge discrepancy was reported between ownership *versus* utilization of insecticide-treated bed nets (ITNs). To acquire the benefits of ITNs, households need to use and not merely own them. The objective of this study was to characterize the pattern of, and assess factors related to ITN use in one village in south Ethiopia.

**Methods:**

A prospective cohort study involving 8,121 residents (in 1,388 households) was carried out from April 2009 to April 2011 (101 weeks). Every week, individuals were asked whether they slept under an ITN the night before the interview. Descriptive statistics was used to report the availability and use of ITN. A negative, binomial, probability, distribution model was fitted to find out significant predictors of ITN use. Reasons for not using ITN were summarized.

**Results:**

The total number of ITNs available at the beginning of the study was 1,631 (1.68 ITNs per household). On week 48, 3,099 new ITNs (PermaNet2.0) were distributed freely (2.3 ITNs per household). The number of households who received at least one new ITN was 1,309 (98.4%). The percentage of children <5 years and pregnant women not using ITNs exceeded that of other adults. The mean (range; SD) ITN use fraction before and after mass distribution was 0.20 (0.15-0.27; 0.03) and 0.62 (0.47-0.69; 0.04), respectively. Before mass ITN distribution, the most frequent reason for not using ITN was having worn out bed nets (most complained the bed nets were torn by rats); and after mass ITN distribution, it was lack of convenient space to hang more than one ITN. Males, younger age groups (mainly 15–24 years) and those living away from the vector-breeding site were less likely to use ITN.

**Conclusions:**

The ITN use fraction reached to a maximum of 69% despite near universal coverage (98.4%) was achieved. Gender, age differences and distance from vector breeding site were associated with ITN use. Strategies may need to be designed addressing disproportions in ITN use, lack of convenient space to hang more than one ITN (for those receiving more than one), and measures to prolong usable life of ITNs.

## Background

Insecticide-treated bed nets (ITNs) are the tools of malaria control and prevention [1]. The impact of ITNs on reducing malaria episodes is well documented [2,3]. Use of ITNs is one of the major vector control measures in Ethiopia. More than 20 million ITNs were distributed between 2005 and 2007, enabling 68% of the households living in malaria-endemic areas to own at least one ITN [4]. The recent national strategic plan targets that at least 80% of people at risk of malaria shall use ITNs properly and consistently, whereby, 100% of households in malaria-endemic areas should own one ITN per sleeping space by the year 2015. The country aims at malaria elimination in areas with historically low malaria transmission, while achieving near zero malaria transmission in the remaining malarious areas [5]. To achieve such a goal, better understanding of utilization of prevention and control tools, mainly ITNs, is essential.

A huge discrepancy was reported between ownership *versus* use of ITNs [1]. Studies quantified this difference as 95% *vs* 59% (Kenya) [6], 70% *vs* 53.1% (Nigeria) [7] and 90% *vs* 77% (Tanzania) [8]. Misconceptions about prevention of malaria, discomfort, perceived low mosquito density, inconvenience to hang the nets, place of residence, economic and educational background, age and gender differences, and colour of nets were among the reported reasons related to ITN utilization [9-15].

To acquire the benefits of ITNs, households need to use, not merely own them [1]. This calls for a need to study ITN use. This study aims to characterize the pattern of ITN utilization and determine associated factors in one malaria-endemic village in south Ethiopia.

## Methods

### Setting

Chano Mille village is one of the rural, malarious areas near Arba Minch town, 492 km south-west of Addis Ababa. The altitude is 1,206 m above sea level. The village was selected purposively to study malaria epidemiology in detail in the presence of favourable malaria vector breeding site. The presence of Lake Abaya to the south-east of the village resulted in intense malaria transmission since the shore of the lake favoured malaria vector breeding. The incidence rates of falciparum and vivax malaria in the village were 22.9 and 22.2 per 1,000 persons per year, respectively. The distance of the household from the shore of the lake, wealth index, age and gender were found to be significant predictors of malaria infection. In the two-year study period, the government had undertaken indoor residual spraying with insecticides (twice) and mass free ITN distribution (once) as prevention and control measures [16,17].

### Study design and data

This was a prospective cohort study that involved all residents of the village. The total number of households was 1,388 and the total number of individuals followed for 101 weeks (from April 2009 to April 2011) was 8,121. Every week, individuals were asked whether they slept under an ITN the night before the interview; and if they did not use the ITN, open-ended question was used to ask the reason why. To maintain a gap of six days between the visits, households were visited on the same day each week. A census was carried out three times to update the denominator: at the start of the study, on week 50, and at the end of the study. For the first four weeks, ITN use data were collected considering vulnerable groups, including children under five years and pregnant women. After week 5, all residents of the village were considered and the name of the individual who slept under an ITN was recorded. Weekly ITN use fraction was calculated by taking the number of individuals who slept under an ITN as numerator and the total population of the week as denominator; this was done for different gender and age categories as well. The number of weekly follow-ups in which ITN use was reported was calculated for each of 8,121 individuals. The number of bed nets available at each household was recorded at the beginning. In addition, after free mass distribution of ITNs, which was carried out by the government on week 48, ITN coverage survey was carried out on week 50. During the ITN coverage survey, the households were asked if they had usable ITNs in addition to the new ones; and when available, these ITNs were considered old-functional.

The data collectors were recruited from the village having college level diploma.

### Data analysis

Summary statistics were used to report the number of bed nets (new and old-functional) available in each household, and the proportion of children aged less than five years, and pregnant women that did not use ITNs. Likewise, the median number of weekly follow-ups in which ITN use was reported was calculated for different population sub-groups. A summary was provided on the reported reasons for not using ITN.

The count data on the number of weekly follow-ups, in which ITN use was reported, was over dispersed while Poisson regression was fitted. The ratio of the deviance over the degree of freedom was 24.4. This value became 1.6 with a negative binomial probability distribution model. As the later model handled the overdispersion problem (since the value was very close to 1), a negative binomial regression model was fitted to the data. The number of weeks an individual was observed was set as a scale weight variable. A fixed value of 1 was used as a scale parameter method and robust estimator was used for the covariance matrix. Exponential parameter estimates were interpreted as incidence rate ratios (IRR). The 95% confidence intervals (CI) for the IRR were also reported. Gender, age, education of the household head, wealth tertiles and distance (in km) from vector breeding site were considered as determinants for ITN use. To construct wealth index, principal component analysis (PCA) was used. The variables included were presence of electricity, watch, TV, radio, mobile phone, refrigerator, separate room used for kitchen, bicycle, agricultural land, livestock, account in bank or credit association and latrine facility. In addition, the main materials of the floor, wall and roof were considered. The details of wealth index construction are reported elsewhere [16]. Distance of each household (in km) from the identified vector breeding site was calculated using proximity analysis tool of ESRI ArcMap 9.3 (Redlands, CA, USA). Statistically significant independent variables during bivariate analyses were used to construct the multivariate model. Pairwise comparison was done for age categories using sequential Sidak as adjustment for multiple comparisons. PASW 18.0 (Chicago, IL, USA) was used for analysis.

### Ethical clearance

The Southern Nations and Nationalities Regional Health Bureau Ethical Review Committee approved this study. Permission and support letters were obtained from relevant administrative bodies of the area. Informed verbal consent was obtained from each household.

## Results

The total population followed was 8,121 in 1,388 households making the average household size 5.9 persons.

### ITN coverage

The total number of nets available at the beginning of the study was 1,631 (1.68 ITNs per household). All bed nets were PermaNet2.0 and 241 (19.9%) reported that they did not have any bed nets (Table 1).

**Table 1:** Number of insecticide-treated bed nets per household according to the first census

**Number of ITNs per household (n = 1212)**	**Number**	**Percent**
0	241	19.9
1	414	34.2
2	463	38.2
3	85	7.0
4	9	0.7
1-4	971	80.1

According to the ITN coverage survey (carried out on week 50), the households reported that they had 916 old-functional ITNs. On week 48, they received 3,099 new ITNs (PermaNet2.0) for free (2.3 ITNs per household). The number of households receiving at least one new ITN was 1,309 (98.4%), and nearly half, 605 (45.4%) of the households received three ITNs each (Table 2).

**Table 2:** Number of insecticide-treated bed nets according to the second census conducted on week 50

**Number of ITNs available per household**	**Old-functional**^**¥ **^**n = 1,156 households**		**New**^**‡ **^**n = 1,330 households**	
	**Number**	**Percent**	**Number**	**Percent**
0	530	45.8	21^§^	1.6
1	372	32.2	201	15.1
2	223	19.3	468	35.2
3	28	2.4	605	45.5
4	2	0.2	29	2.2
5	0	0.0	5	0.4
6	1	0.1	1	0.1
1-6	626	54.2	1309	98.4

^¥^Distributed three years previously.

^‡^Distributed two weeks previously, the number of households involves both existing and newly added ones.

^§^Four existing households and 17 newly enrolled households.

### ITN use fraction

**Figure 1:**
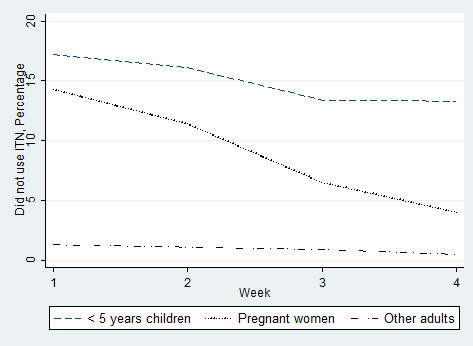
Percentage of < 5 years, pregnant women and other adults not using available insecticide-treated bed nets (in the first four weeks of observation).

In the first four weeks of observation, the percentage of ITN use was determined for vulnerable groups. The data showed that the percentage of under five years children not using ITNs (followed by pregnant women) exceeded that of other adults (Figure 1).

**Figure 2:**
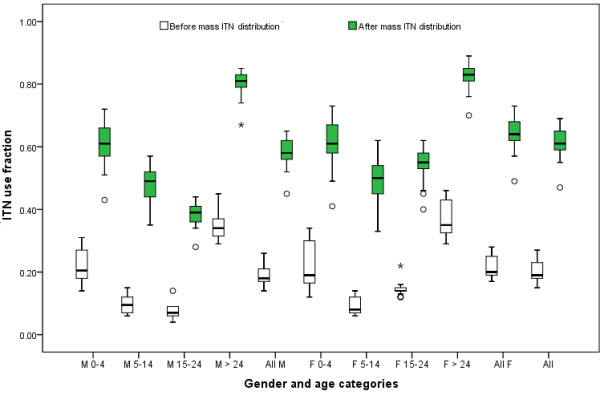
**Distribution of insecticide-treated bed net use fraction according to gender and age categories (in year) before and after mass insecticide-treated bed net distribution.** M and F refer to males and females, respectively.

The mean (range; SD) ITN use fraction before and after mass distribution was 0.20 (0.15-0.27; 0.03) and 0.62 (0.47-0.69; 0.04), respectively. The distribution of ITN use fraction is presented in Figure 2.

**Figure 3:**
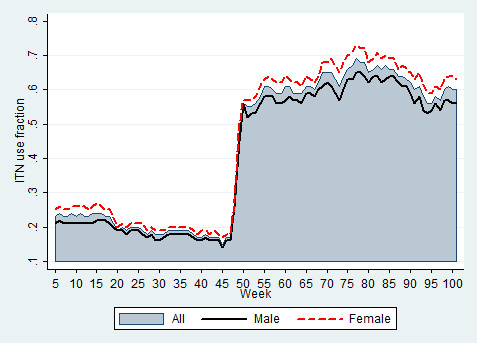
Insecticide-treated bed net use fraction by gender.

**Figure 4:**
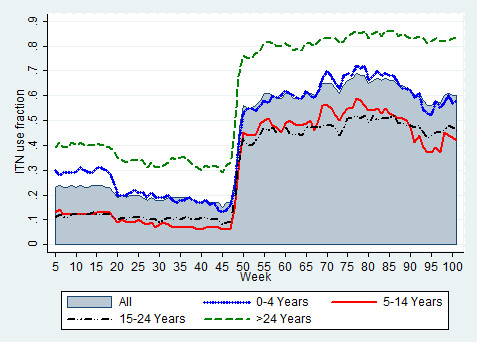
Insecticide-treated bed net use fraction by age group.

The significant increase in ITN use fraction after week 48 indicates the time of free mass ITN distribution. In general, the proportion of females using bed nets exceeded that of males in all weeks of observation (Figure 3). The proportion of adults aged above 24 years (followed by children less than five years old) using ITNs surpassed all other age categories (Figure 4).

**Figure 5:**
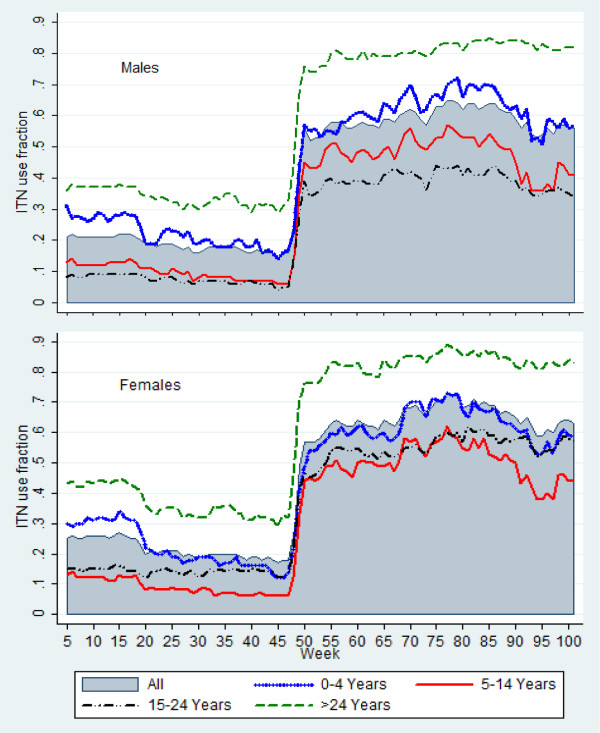
Insecticide-treated bed net use fraction by gender and age group.

Figure 5 shows the ITN use fraction according to age groups and gender. The gap in the level of ITN use fraction between age groups five to 14 and 15 to 24 became wider after free mass ITN distribution, mainly for males (less use among aged 15 to 24 years); and among females, more use was recorded among aged 15 to 24 years for most of the weeks. For most of the weeks, females under five years had less ITN use fraction compared to the whole population, which was not the case for their male counterparts.

### Reasons for not using insecticide-treated bed nets

The most frequent reason for not using an ITN was having worn-out bed nets (most complained that the bed nets were torn by rats). Some did not hang the bed nets because of being dirty, changes in bed arrangement, unsuitable housing structure and considering alternatives such as insecticide sprays. The other reasons were absence of mosquitoes within the house, sleeping in the farm and presence of social gatherings because of death of family member. Some reported that they provided the bed nets to their children as they sent them to other places for schooling. Some reported discomfort (feeling warmth) while sleeping under the net and a few reported that they used the bed nets as curtain for traditional pit latrines. Meanwhile, after mass ITN distributions, the most frequent reason for not using bed nets was lack of convenient space in the house to hang more than one bed net.

### Total number of weekly follow-ups in which insecticide treated bed net use was reported

The median number of weekly follow-ups in which mosquito net use was reported over 97 weeks of observation was higher for females, the age category >24 years, the poor, and residents having secondary level education (Table 3).

**Table 3:** Median number of weeks in which insecticide treated bed net use was reported over 97 weeks of observation

**Variables**		**N**	**Median **^**§**^
Gender	Males	4227	26
	Females	3894	34
Age in years	< 5	1067	39
	5–14	2175	25
	15–24	2321	7
	>24	2558	52
Wealth tertiles	Poor	2671	35
	Medium	3168	32
	Rich	2282	19
Education of the household head	No education	4351	25
	Primary	2050	32.5
	Secondary	1616	41
	Above secondary	104	29.5
All		8121	30

^§^Total number of weeks = 97.

### Predictors of total number of weekly follow-ups in which insecticide treated bed net use was reported

Wealth tertiles and level of education of the household head did not show statistical significance during bivariate analysis. Controlled for other variables, the rate of total number of weeks spent under ITNs was less by 10% among males. This rate was 54% less for age category 15 to 24 years compared to adults >24 years old. Meanwhile, this rate decreased by 19% for each km distance away from the vector breeding site (Table 4).

**Table 4:** Factors associated with insecticide-treated bed net use

**Variable (n = 8121)**		**Total number of weeks spent under ITNs**	
		**Crude IRR (95% CI)**	**Adjusted IRR (95% CI)**
Gender: Male		0.9(0.87-0.93)*	0.9(0.87-0.93) *
Age in years^‡^	< 5	0.71(0.68-0.74) *	0.71(0.68-0.74) *
	5–14	0.51(0.49-0.53) *	0.51(0.49-0.53) *
	15–24	0.46(0.44-0.48) *	0.46(0.43-0.48) *
Wealth tertiles^¥^	Poor	1.04(0.99-1.09)	NA
	Medium	1.04(0.99-1.09)	NA
Education of the household head^§^	No education	0.9(0.77-1.05)	NA
	Primary	0.99(0.85-1.17)	NA
	Secondary	1.12(0.96-1.31)	NA
Distance (km) from vector breeding site		0.84(0.8-0.88) *	0.81(0.77-0.85) *

^‡^Reference category: >24 years.

^¥^Reference category: Rich.

^§^Reference category: Above secondary.

*Significant at 0.05 level.

NA: Not applicable.

The pairwise comparison showed that children under five years old spent more number of weeks under the ITNs compared to age categories five to 14 and 15 to 24 years but less compared to age category >24 years. The least number of weeks spent under ITNs was observed among 15 to 24 years old compared to all other age categories (Table 5).

**Table 5:** Pairwise comparisons of estimated marginal means of age categories based on the original scale of total number of weeks spent under insecticide-treated bed nets

	**Category**		**Mean difference (A-B)**	**P value (Sequential Sidak)**
	**A**	**B**		
Age in years^‡^	<5	5-14	Positive	<0.001
		15-24	Positive	<0.001
		>24	Negative	<0.001
	5-14	15-24	Positive	0.002
		>24	Negative	<0.001
	15-24	>24	Negative	<0.001

^‡^Overall test results: Wald Chi-Square = 1036.68 and P value < 0.001.

## Discussion

Coverage of new ITN distribution was 98.4% and the maximum ITN use fraction was 69%. The percentage of under five years and pregnant women not using ITNs exceeded that of other adults. Being male, younger, and living farther from the vector breeding site were factors associated with less frequent use of ITNs. Residents in the age range 15 to 24 years were the least users of ITNs. Lack of convenient space to hang the ITN was the prominent reason for not using ITN, despite its availability.

ITN use fraction was calculated based on self-report. It may not be possible to avoid bias with self-report. However, listing the names of household members who slept under ITN the night before the interview was considered to be better than asking a Yes/No question. A similar approach was used in previous studies [10]. The fact that the ITN use fraction did not reach 100% (the maximum was 69%) after mass distribution of ITNs was reassuring in that social-desirability bias did not overwhelm this study.

Distance from the vector breeding site affected use of ITNs. This may support the notion that ITN use is associated with risk perception [10]. This was also indicated by the finding that the number of malaria episodes decreases in the household farther from the vector breeding site [16,17], which might have compelled residents who lived away from the vector breeding site to perceive lower risk of disease and to use ITNs less frequently.

A recent paper showed that before mass ITN distribution, the risk of *falciparum* malaria was higher in the age category <15 years compared to 15 to 24 years. However, this risk shifted to the age category 15 to 24 years after mass ITN distribution [16]. This could be explained by the significant differences in frequency of ITN use among different age categories, whereby less frequent use of ITNs was observed in the category 15 to 24 years. Though this less frequent use (in 15 to 24 years category) had existed before mass ITN distribution, the increased frequency of ITN use among the younger age categories (<15 years) after mass ITN distribution could move the risk towards 15 to 24 years category.

Educational status and wealth index did not significantly affect ITN use in this study. Some studies reported similar findings [7,18,19], while the others showed significant associations between socio-economic factors and ITN use [11,12]. The presence of prominent vector breeding site yielding varying risk to the households in the study area was worthy of note since the households located closer to the vector breeding site reported more frequent use of ITNs, implying influence of nuisance mosquitoes and/or risk perception might have outweighed factors such as education and wealth, with regard to sleeping under ITNs.

The first four weeks of observation showed adults using ITNs more than the vulnerable groups: under five years and pregnant women. Similarly, during the remaining 97 weeks of follow up, adults used ITNs more than the younger (<24 years) residents and this was not expected. This might also have resulted in lower incidence rate of *falciparum* malaria among adults since ITN use at individual level was reported to be protective. Similar speculation could be derived for male study participants, as males used ITNs less frequently and suffered more from *falciparum* malaria [16].

The most frequent reason for not using ITNs before mass distribution was having worn-out bed nets, mainly because the ITNs were ragged by rats. This implies the need to integrate rodent control measures with ITN distribution in areas with a similar problem in order to lengthen the usable life of ITNs, considering cost implications in distributing free ITNs more often.

Except those who did not receive new ITNs, no household reported inadequacy of number of ITNs received during mass distribution; however, frequency of ITN use did not reach to the coverage. The maximum ITN use fraction was 69% while coverage was 98.4%. Studies indicated such discrepancies between bed net coverage and use [6-8]. Quantitative data showed individual differences in frequency of bed net use including age, gender and risk perception. Meanwhile, according to the responses to the open-ended question, the most frequent reason for not using bed nets, while at least one was available, was lack of convenient space to hang the bed nets. This was also the case in the review made on reported reasons for not using ITNs [10]. This implies mere calculation of ratio of number of household members to bed nets (while distributing the nets), without considering the housing structure or helping the household to hang the desired number of bed nets, would not bring this prevention and control measure to the intended level of efficiency and effectiveness. Meanwhile, the unusual practice of using bed nets for other purposes, including as curtains for traditional pit latrines (though not frequently reported) should be properly addressed.

## Conclusions

The ITN use fraction reached to a maximum of 69% despite near universal coverage. Residents aged above 24 years used ITNs more than the younger age categories, while those aged 15 to 24 years and males were the least users. Households that were distant from the main vector breeding site were less likely to use ITNs. After mass ITN distribution, lack of convenient space to hang more than one bed net was the most frequently reported reason for not using ITNs. Use of ITNs as malaria prevention and control may benefit from combinations of strategies to improve ITN use among the younger age groups, to ensure that each household managed to hang all the bed nets provided for free, and to incorporate measures (such as rodent control) that could prolong the usable life of bed nets. To better understand the reasons for not using ITN, well designed qualitative research approach should be considered.

## Competing interests

The authors declare that they have no competing interests.

## Authors’ contributions

EL conceived the study, collected, analysed and interpreted data, and prepared the draft manuscript. KT participated in data collection. BL conceived the study, interpreted data and helped to draft the manuscript. All authors read and approved the final version of the manuscript.
